# Increasing competition in pharmaceutical tenders: Patents, treatment guidelines, and centralised procurement of Hepatitis C drugs in Brazil

**DOI:** 10.1080/17441692.2026.2708415

**Published:** 2026-07-23

**Authors:** Eduardo Mercadante

**Affiliations:** a Department of International Development, London School of Economics and Political Science (LSE), London, United Kingdom

**Keywords:** Drugs, pharmaceutical patents, public procurement, Hepatitis C, Brazil

## Abstract

This article investigates how therapeutic substitutability and patent exclusivity influence the centralised procurement of Hepatitis C drugs in Brazil. Combining data on treatment guidelines, registered products, and patent applications, this article analyses how the government can increase competition in tenders. Since 2019, the public procurement rationale has shifted from the cost-effectiveness of discrete products to simply buying the cheapest among treatment alternatives that are virtually equal in effectiveness, with originators of different patented drugs competing against each other. Moreover, rigorous examination of patent applications and robust opposition mechanisms led to limited patent exclusivity in some cases, allowing generic manufacturers to compete with the originators of their drugs and other drugs. The findings are discussed based on a matrix of competition scenarios and the literature on patent exclusivity, drug prices, and public procurement. The strategy of having both on- and off-patent competition in the same tender significantly increased the government’s bargaining power and reduced drug prices. However, market concentration has limited this strategy. While helpful, local production and price control policies were less relevant than suggested. Therefore, this article provides lessons on how to combine different public policies to increase bargaining power and thereby promote access to high-quality treatments.

## 1. Introduction

Patents are not a binary mechanism in which their presence or absence leads to full or no exclusivity. Patents are less effective if the product itself is not covered, and competitors develop alternative manufacturing methods that circumvent the protection. Even if the product is protected, alternative treatments might create possibilities for competition. Thus, the effect of patents on pharmaceutical competition depends on the strength, scope, and duration of the protection and is influenced by factors such as therapeutic substitutability. This article investigates how patent exclusivity and therapeutic substitutability influence the competition in centralised pharmaceutical tenders.

In this article, a ‘drug’ is an active pharmaceutical ingredient (API), a ‘product’ is a version of a drug manufactured for marketing, and a ‘regimen’ is a unit of treatment. When all drugs used to treat a disease are patented, and the respective regimens differ significantly in effectiveness, governments tend to negotiate purchases independently with each manufacturer, also called ‘originator,’ when deciding which product to purchase (Syversen et al., 2024). When a drug is not under effective patent protection, off-patent tendering is a well-established strategy to purchase products at lower prices due to generic competition (Maniadakis et al., 2018; Parmaksiz et al., 2022). Recently, some countries have begun exploring on-patent tendering strategies, which include different patented drugs when the alternative regimens are comparable in effectiveness (Barrenho et al., 2023; Wouters et al., 2019). Combining the lack of patent exclusivity for certain drugs with the therapeutic substitutability of different regimens, tenders may include both originators and generic manufacturers of different products. In other words, a single tender may have both on- and off-patent competition. This article investigates these four scenarios by drawing on Brazil’s experience treating patients with the Hepatitis C virus (HCV).

The introduction of direct-acting antiviral (DAA) drugs revolutionised HCV treatment, offering much quicker, simpler regimens with very high cure rates and fewer side effects (Lobato et al., 2019; Mathur et al., 2018; Sulkowski et al., 2021; Zoratti et al., 2020). Also, there are multiple highly effective regimens, increasing access and success rate. Notwithstanding the advances in HCV treatment, there is substantial controversy around DAA drugs, especially sofosbuvir. When introduced in 2014, sofosbuvir became the most expensive pill on the market, creating significant financial pressure even for wealthy countries and raising questions about its patentability, which led to numerous disputes between the originator, Gilead, and other originators, generic manufacturers, and civil society organisations (Bourgeron & Geiger, 2022; Douglass et al., 2018). This controversy was fuelled by sofosbuvir being the most important drug for HCV, included in many combination regimens, and by the volume of public funds that went into its development (Barenie et al., 2021). In response, Gilead issued several voluntary licences, but opposition remained strong in countries excluded from such agreements, leading to many applications being rejected, patents being granted with reduced claims, and threats of compulsory licences being made, to which Gilead responded by extending the voluntary licences to other countries (Douglass et al., 2018).

Brazil is an interesting case study because it is a middle-income country with a high HCV incidence that is not covered by Gilead’s licensing agreements (Douglass et al., 2018). It has also played a leading role in raising global awareness of viral hepatitis and has vowed to cure any Brazilian resident infected with HCV through the Unified Health System (SUS), forcing the government to optimise expenditure (Chaves et al., 2017; Davidian & Fonseca, 2022; WHS – World Hepatitis Summit, 2017). However, detection and treatment have fallen short of what the Ministry of Health (MoH) deemed necessary to treat the estimated 632,000 cases of HCV infection (Benzaken et al., 2019; MoH – Ministry of Health, 2024a).

This article analyses four scenarios of competition in the centralised procurement of HCV drugs based on the effectiveness of alternative regimens recommended by the MoH since 2015, on the originator and generic products approved by the National Health Surveillance Agency (Anvisa), and on each drug’s level of patent exclusivity. The findings show that the government could increase competition in tenders because newer DAA-based regimens were highly comparable in effectiveness, allowing it to purchase the cheapest option. Competition was further increased by gaps in patent exclusivity, which allowed originators and generic manufacturers to compete in the same tender, increasing the government's bargaining power and leading to the lowest drug prices. Incentives to local production helped this strategy by promoting the launch of generic versions of drugs with limited patent exclusivity. Conversely, price control policies did not affect centralised procurement, as contracts were negotiated well below the price caps. Furthermore, the positive effects from tenders were limited by market concentration when drugs were removed from the guidelines, when manufacturers discontinued them, or when suitable competitors refused to place offers.

The following sections present the Methods for this study, the data on treatment guidelines, market approvals, and patent landscapes, and timelines combining these data for each HCV drug. Next, I analyse the centralised procurement history. Then, I discuss the findings, drawing on the literature on patent exclusivity, drug prices, and public procurement. Finally, the Conclusion presents the key lessons and recommendations from this case study.

## 2. Methods

The goal of this article is to investigate how the Brazilian government’s bargaining power in centralised procurement of HCV drugs was influenced by the drugs’ therapeutic substitutability and patent exclusivity. The analysis is restricted to purchases from 2015, since in that year the MoH introduced the second generation of DAA drugs, revolutionising HCV treatment with higher cure rates and lower side effects.

**Figure 1.**
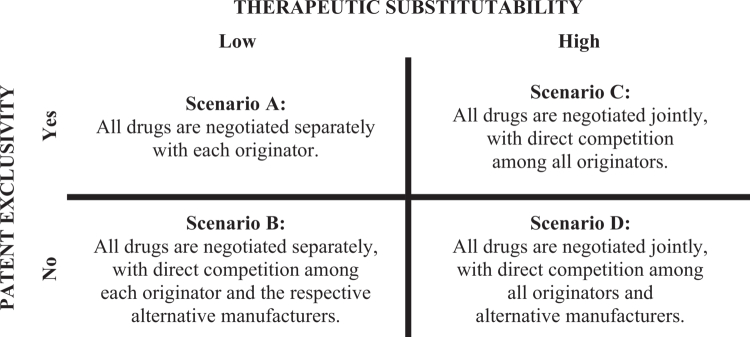
Scenarios of competition based on patent exclusivity and therapeutic substitutability.

The negotiations in centralised procurement processes are analysed using a matrix of four competition scenarios, as presented in Figure 1. In scenario A, all drugs are under patent exclusivity and differ significantly in effectiveness, so separate contracts must be negotiated with originators. In scenario B, different drugs are therapeutically distinct, but one drug lacks patent exclusivity, so its originator and generic manufacturers may be placed in direct competition—though still separately from other drugs. In scenario C, all drugs are patented and highly comparable in effectiveness, allowing direct competition among originators. In scenario D, effectiveness is highly comparable, and at least one drug is not under exclusivity, allowing direct competition among all originators and generic manufacturers.

This analysis requires combining four types of data: (1) treatment guidelines; (2) marketing approvals; (3) patent applications; and (4) procurement records. The following subsections explain the data collection and analysis strategies for these four types of data and present this article's limitations.

### 2.1. Treatment guidelines

The MoH periodically updates HCV treatment guidelines to include the most effective drugs that have already obtained market approval and been incorporated into SUS (MoH – Ministry of Health, 2011, 2015, 2017, 2019, 2022a, 2023). In Section 3, I present data extracted from these guidelines: (1) which drugs were included or excluded from the roll of recommended regimens; (2) specific characteristics of each regimen, such as concentration, form, daily dose, and treatment period; (3) if treatments were restricted to specific types of patients or offered universally; and (4) the MoH’s assessment of therapeutic substitutability.

### 2.2. Marketing approval

As the health regulator, Anvisa performs multiple functions in the Brazilian pharmaceutical and healthcare sectors. This article focuses on Anvisa’s General Office of Medicines (GGMED), which is responsible for assessing quality, safety, and efficacy, and on Anvisa’s Drug Market Regulation Chamber (CMED), which sets maximum wholesale and retail prices, as well as the mandatory discount for government procurement of strategic products.

For small-molecule therapeutics, there are three product types. ‘Originator’ products are new molecules registered in Brazil for the first time with proven therapeutic gain, must be covered by at least one Brazilian patent application, and receive a price cap that cannot be higher than the lowest price in 14 reference countries. ‘Generic’ products are identical versions of molecules already registered for a specific therapeutic use, must prove equal bioequivalence and bioavailability as originator products, must be sold under the API rather than any brand name, and must have caps that are at least 35% lower than those of originator products. ‘Branded generic’ products have the same technical requirements as generics but may be sold under brand names and can receive caps lower than or equal to those of originator products. Anvisa calls these categories ‘new,’ ‘generic,’ and ‘similar’ (Anvisa – National Health Surveillance Agency, 2022).

Once products pass GGMED’s assessment and CMED sets price caps, Anvisa issues a 10-year marketing approval, which may be renewed unless manufacturers discontinue the products. In Section 4, I present data extracted from Anvisa’s database: product names; manufacturers; product types; whether products were manufactured locally or imported; registration dates; and discontinuation dates, if applicable (Anvisa – National Health Surveillance Agency, 2024).

### 2.3. Patent applications

The strategy for mapping the landscape of HCV drug patent applications in Brazil included only applications covering compounds or their combinations listed in the guidelines. Since there is no central Brazilian registry connecting patent applications to drugs, I drew on multiple sources: the United States Food and Drug Administration’s Orange Book, which connects approved drugs and granted patents on compounds of synthetic drugs, their indications, or methods of use (FDA – Food & Drug Administration, 2023); the Medicines Patent Pool’s Medicines Patent and Licence (MedsPaL) database, which lists key applications for specific drugs (MPP – Medicines Patent Pool, 2023); previous HCV patent landscapes (UNITAID, 2015a, 2015b, 2017a, 2017b, 2017c; WHO – World Health Organisation, 2016); and the MoH’s requests for fast-tracked prosecution of strategic applications (INPI – National Institute of Industrial Property, 2019b).

In November 2024, I searched the Buscaweb database of the Brazilian patent office, the National Institute of Industrial Property (INPI), for applications corresponding to those listed by these sources, using the Patent Cooperation Treaty (PCT) numbers (INPI – National Institute of Industrial Property, 2024). Next, I investigated prosecution outcomes, oppositions, litigation, appeals, and the use of divisional applications and fast-track examination.

The results of this patent landscape are presented and analysed in Section 5. Next, Section 6 presents timelines for each drug from 2015, indicating when the different product types were registered and discontinued, when the drugs were included in the treatment guidelines, and their patent protection status. For that, I considered three patent protection periods. While applications are pending, applicants have ‘provisional rights’ because they may claim retroactive compensation after the grant for infringement that occurred during prosecution, which may create *de facto* exclusivity if launching alternatives is risky (grant is likely) and costly (effective enforcement). If the patents are granted, applicants gain ‘patent rights,’ or *de jure* exclusivity. If applications are rejected or abandoned, or when the patent term ends, inventions enter the ‘public domain.’ Based on the timelines, I explain the circumstances that allowed generic competition for certain drugs but not others.

### 2.4. Procurement records

Despite the MoH centralising procurement since 2006, other private and public entities may purchase HCV products, including when mandated by courts (Chaves et al., 2017; Davidian & Fonseca, 2022). Patients often seek court mandates when products are too costly or in experimental stages (Caetano et al., 2022; Lamprea, 2017). In 2016, these purchases represented 7% of public expenditure on drugs, with sofosbuvir having one of the highest shares (Vieira, 2020).

This article’s main analysis focuses on centralised tenders by the MoH. Some explanations about the tenders are due. Each tender is divided into groups based on HCV genotypes and patients’ conditions. The MoH sets individual maximum prices for each group that cannot exceed the price caps established by CMED/Anvisa. Only products approved by Anvisa may be offered. Manufacturers and distributors of the same drug may place competing offers. Winning offers may be split into multiple contracts. Any participant may file an appeal with the auctioneer, and records are available in the ComprasNet database (Brazil, 2024b). Based on these data, Section 7 analyses the history of centralised procurement by the MoH since 2015, excluding court-ordered purchases, using data extracted from the Transparency Portal and the ComprasNet database (Brazil, 2024a, 2024b).

### 2.5. Limitations

The main limitations of this article stem not from the issues with data availability but rather from characteristics of the case study. I identified all drugs recommended for HCV treatment, all products approved for marketing, all patent applications filed in Brazil, and all centralised procurement processes by the MoH. However, the government centralises procurement to make as few purchases as possible, thus maximising volumes and minimising prices. Therefore, the number of purchases is insufficient for a generalisable statistical analysis of the impact of therapeutic substitutability and patent exclusivity on drug prices. Furthermore, Section 7 shows that differences in patent exclusivity among drugs result from manufacturers’ commercial strategies and the scope of patent applications, not from the government treating specific drugs differently.

Given these limitations, this article employed a mixed-methods approach in which quantitative results on procurement prices are analysed vis-à-vis qualitative aspects of the type of negotiation enabled by the levels of therapeutic substitutability and patent exclusivity. The findings should thus be interpreted as indicative of the potential effect of this procurement strategy on competition, rather than as a deterministic measure of its impact on prices.

## 3. The history of HCV treatment in Brazil

When DAAs were introduced in 2013, treatment was limited to severe cases due to the lack of experience with these drugs and budget restrictions, and purchase decisions were based on cost-effectiveness since the regimens produced different results. From 2015, the MoH introduced the most effective DAA drugs, which are the focus of this article. Given the better outcomes with newer treatments and the commitment to eliminating HCV as a public health threat by 2030, the MoH expanded treatment to moderate cases in 2017. Table 1 presents all DAA-based regimens recommended since 2015. I use a plus sign for drugs combined into a single tablet and a slash for different tablets that must be taken together, focusing on the treatment of adults without coinfections who have never taken DAA drugs.

**Table 1.** List of DAA-based HCV treatment regimens in Brazil since 2015.

Regimens	Concentration	Form	Treatment		Guidelines				
			Daily dose	Duration (weeks)	2015	2017	2019	2022	2023
elbasvir/grazoprevir	50mg/100mg	Tablet	1	16			X		
glecaprevir/pibrentasvir	100mg/40mg	Tablet	1	8			X	X	
ledipasvir/sofosbuvir	90mg/400mg	Tablet	1	12			X		
sofosbuvir+daclatasvir	400mg + 60mg	Tablets	1 + 1	12	X	X	X	X	X
sofosbuvir+simeprevir	400mg + 150mg	Tablets	1 + 1	12	X	X			
sofosbuvir+glecaprevir/pibrentasvir	400mg + 100mg/40mg	Tablets	1 + 1	12					X
velpatasvir/sofosbuvir	100mg/400mg	Tablet	1	12			X	X	X
veruprevir/ritonavir/ombitasvir +dasabuvir (3D)	75mg/50mg/12.5mg + 250mg	Tablets	2 + 2	12		X			

The most important change occurred in 2019 when the MoH declared that the high level of ‘therapeutic effectiveness, measured by the sustained virologic response (SVR), is absolutely comparable across all proposed regimens when similar clinical situations are evaluated’ (MoH – Ministry of Health, 2019, p. 41). Moreover, the MoH determined that treatment should be universally accessible and that the purchasing rationale should shift from cost-effectiveness to cost-minimisation, i.e. buying the cheapest option for similar cases. This decision follows recommendations from the World Health Organisation (WHO) and the evidence from clinical studies (Lobato et al., 2019; Mathur et al., 2018; Sulkowski et al., 2021; Zoratti et al., 2020).

In 2022, the MoH limited purchases to WHO-recommended pangenotypic regimens, which may be given to patients with similar conditions regardless of HCV genotypes, making treatment cheaper, simpler and faster: sofosbuvir+daclatasvir, glecaprevir/pibrentasvir and velpatasvir/sofosbuvir (WHO – World Health Organisation, 2024; Zoratti et al., 2020). In 2023, the MoH defined sofosbuvir+daclatasvir or velpatasvir/sofosbuvir as the first treatment options, recommending sofosbuvir+glecaprevir/pibrentasvir in case of therapeutic failure.

## 4. Products registered in Brazil for HCV treatment

In Table 2, I present marketing approval data for 15 HCV products. Apart from the originator products, generics have been approved for two drugs. In many countries, this can only be done once the drugs are no longer under patent protection (Raju, 2022; Son et al., 2018). However, Brazil allows the registration of generic versions of drugs under patent exclusivity to avoid delaying their launch.

**Table 2.** Products for HCV treatment with marketing authorisation by Anvisa.

Product	Drugs	Lab	Type	Production	Registered	Discontinued
Daklinza	daclatasvir	BMS	Originator	Imported	06/01/2015	11/12/2020
daclatasvir dihydrochloride	daclatasvir	Blanver	Generic	Imported	05/12/2022	
daclatasvir dihydrochloride	daclatasvir	Fiocruz	Generic	Imported	26/02/2024	
Zepatier	elbasvir/grazoprevir	MSD	Originator	Imported	04/12/2017	30/09/2022
Maviret	glecaprevir/pibrentasvir	AbbVie	Originator	Imported	16/04/2018	
Harvoni	ledipasvir/sofosbuvir	Gilead	Originator	Imported	04/12/2017	
Olysio	simeprevir	J&J	Originator	Imported	11/03/2015	17/01/2019
Sovaldi	sofosbuvir	Gilead	Originator	Imported	30/03/2015	
sofosbuvir	sofosbuvir	Blanver	Generic	Local	21/05/2018	
sofosbuvir	sofosbuvir	Fiocruz	Generic	Local	02/07/2018	
Sophir	sofosbuvir	Blanver	Branded generic	Local	29/04/2019	13/05/2021
sofosbuvir	sofosbuvir	EMS	Generic	Local	23/11/2020	
sofosbuvir	sofosbuvir	Furp	Generic	Local	29/03/2021	
Epclusa	velpatasvir/sofosbuvir	Gilead	Originator	Imported	25/06/2018	
Viekira	3D	AbbVie	Originator	Imported	22/04/2015	20/02/2020

Note: I considered the permanent discontinuations published by the MoH in the Union’s Official Gazette.

All generics were developed by Productive Development Partnerships (PDPs). Essentially, PDPs are agreements where the MoH promises a share of its purchases to a private firm that voluntarily licences a strategic technology to a national public laboratory. PDPs may involve an international firm transferring the technology, a national firm acting as an intermediary transferor, a public laboratory as the final transferee, and a national pharmochemical firm internalising the API. All members of the PDP must register their versions of the drug (Fonseca & Shadlen, 2026; MoH – Ministry of Health, 2024b; Pimentel, 2018). For example, the Egyptian company Pharco is transferring daclatasvir to Blanver and the Oswaldo Cruz Foundation (Fiocruz), while Microbiológica is internalising the API.

Since Anvisa may approve a generic version while the drug remains patented, one cannot infer the patent status from approved products. In the following two sections, I map the landscape of Brazilian HCV drug patents and investigate whether the patent status influenced the registration of generic versions for only two drugs or the discontinuation of four originator products and the branded generic sofosbuvir.

## 5. The Brazilian patent landscape of HCV drugs

As of November 2024, there were 89 Brazilian applications covering the 15 HCV drugs, as shown in Table 3. For ritonavir, only applications covering the 3D regimen were included, since it is an older drug first approved for other uses and the MoH reported that there were no pending applications or valid compound patents when the regimen was introduced (INPI – National Institute of Industrial Property, 2019b). The considerable difference in the number of applications covering sofosbuvir, compared to all other drugs, is due to a much more frequent use of divisionals. Except for two rejections pending appeal, all applications have reached an outcome: 32% granted, 30% rejected, and 38% abandoned.

**Table 3.** Prosecution history of patent applications related to HCV drugs in Brazil.

Drug	Applications^(1)^		Opposition		Appeal	Litigation	Fast-track	Outcome		
	Total	Divisionals	Anvisa	Others				Granted	Rejected	Abandoned
daclatasvir	12	0	5	1	0	0	8	3^(2)^	2	7
dasabuvir	7	0	1	0	0	0	0	1	1	5
elbasvir	3	0	0	0	0	0	0	1	0	2
glecaprevir	9	0	1	0	1	0	0	3	3^(3)^	3
grazoprevir	6	0	1	0	0	0	0	1	1	4
ledipasvir	9	2	4	0	1	0	5	5	0	4
ombitasvir	5	0	0	0	0	0	0	2	0	3
pibrentasvir	9	0	0	0	1	0	0	3	3^(3)^	3
ritonavir	3	0	0	0	0	0	0	0	0	3
simeprevir	16	1	9	0	0	0	10	5^(2)^	3	8
sofosbuvir	27	11	21	18	10	9	18	5	16^(3)^	6
velpatasvir	6	0	2	1	2	1	2	1	2^(3)^	3
veruprevir	5	1	0	0	0	0	0	1	0	4

Note: (1) Some applications covered multiple drugs, so the sum is bigger than the sample. (2) Applicants let all grants lapse. (3) One appeal to rejection is pending.

There was intense opposition to applications, particularly those covering sofosbuvir. Third parties opposed 20 applications and filed lawsuits against four of them. Meanwhile, Anvisa opposed 41 of the 55 applications it examined. Anvisa’s oppositions were filed as part of a system where, between 2001 and 2021, applications covering inventions for human health were examined by both the INPI and Anvisa (Mercadante, 2026; Guimarães, 2019; Sampat and Shadlen, 2017; Shadlen, 2017). This complex, intensely debated system led to Anvisa’s examination being restricted to strategic applications, based on the MoH’s lists of strategic drugs and therapeutic indications, which explains why it examined only 55 applications.

Given the 2030 Plan to end HCV as a public health threat, it is surprising that the MoH requested fast-tracking for just 38 applications covering daclatasvir, ledipasvir, simeprevir and sofosbuvir. Even the Brazilian Supreme Court has urged the MoH to be more proactive in requesting fast-tracking (Ação Direta de Inconstitucionalidade 5.529, 2021). Two other applications were fast-tracked at the applicant's request. One covered velpatasvir, which Gilead claimed an unauthorised third party was exploiting. The other covered velpatasvir/sofosbuvir, with Gilead trying to expedite the decision on their appeal against the rejection.

## 6. The timelines of treatment guidelines, marketing approvals and patent protection

**Figure 2.**
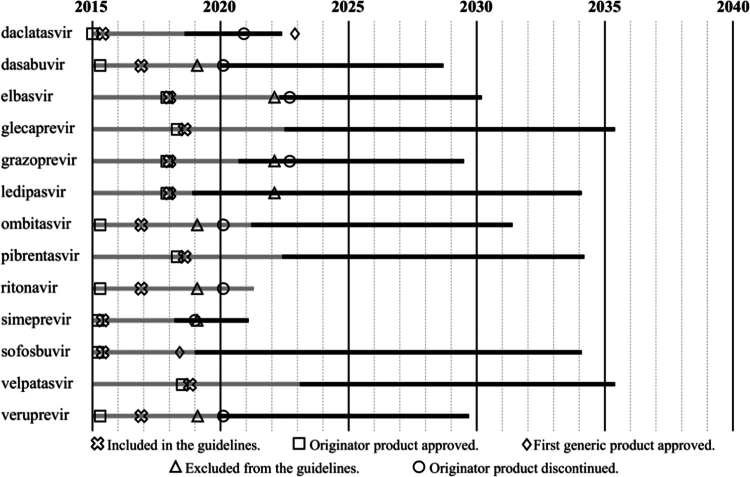
The timelines of HCV drugs in Brazil. Note: The timelines start in 2015 because that is this article’s analysis period, but all drugs had at least one filing before that. The timelines illustrate the periods of patent protection. Grey lines represent the period of provisional rights, when all applications are still pending. Black lines indicate the period of patent rights, when at least one patent has been granted. After that, lines stop to indicate the public domain period, when no valid patent or pending application remains.

In Figure 2, I show the timeline for each drug, indicating when it was recommended, when products were registered and discontinued, and the status of patent protection. A good example of how to read Figure 2 is daclatasvir. In the period of provisional rights, the originator product received marketing approval in January 2015, followed by the drug's recommendation. At that time, competitors could launch generics if they accepted the risk of patent grants and retroactive compensation claims. In August 2018, the first patent was granted, meaning certain aspects of the drug were now under patent rights, allowing BMS to block competitors from exploiting the patented aspects and to claim compensation for infringement that occurred during prosecution. However, BMS discontinued the drug in December 2020 and let all granted patents lapse, thereby entering the public domain in May 2022 and removing all barriers to generic competition. Following this decision, the first generic was approved in December 2022.

Based on the timelines, I investigated why manufacturers discontinued certain products. In most cases, this occurred after the drugs were removed from the guidelines: Zepatier (elbasvir/grazoprevir), Olysio (simeprevir) and Viekira (3D). The MoH also removed ledipasvir/sofosbuvir from the guidelines but recommended using remaining stocks, which explains why Gilead did not discontinue Harvoni. Despite daclatasvir still being recommended, BMS discontinued Daklinza and let all patents lapse as part of a global decision to abandon daclatasvir where it competed with other regimens (MPP – Medicines Patent Pool, 2020). Representatives from Blanver told me that it discontinued Sophir (sofosbuvir) since the MoH would not buy branded generics. Thus, drugs were discontinued when removed from guidelines or due to commercial strategies.

Next, I turned to the registration of generics. The most straightforward case was daclatasvir, since generics were registered after all patents lapsed, despite the drug being recommended. The only other drug with generics was sofosbuvir. There was intense opposition: Anvisa opposed every sofosbuvir application it examined; third parties opposed many applications; the MoH requested fast-track for many applications; and the Brazilian Senate considered issuing a compulsory licence (Senado Federal, 2018). Gilead also fought back: splitting applications to separate controversial aspects and secure some exclusivity, offering counterarguments to oppositions, and appealing or litigating decisions from Anvisa and the INPI. This contentious process resulted in patent protection for sofosbuvir being limited to a chemical intermediate and its uses, derivatives, and formulations, and to its combination with ledipasvir, but not to the compound itself. Generic manufacturers claimed they could use another intermediate, as confirmed by the MoH and the INPI in Senate hearings and in class actions against the sofosbuvir patents (Achcar & Fonseca, 2024; Bermudez, 2018; Fernandes et al., 2024; Senado Federal, 2018). Thus, generics of daclatasvir and sofosbuvir were registered due to a lack of patent exclusivity.

Generics were not registered for other drugs since they were under some exclusivity or not recommended. Many were under patent rights for the compounds: elbasvir, glecaprevir, grazoprevir, ombitasvir, pibrentasvir, and veruprevir. Velpatasvir was under provisional rights for the combination with sofosbuvir, pending an appeal against the rejection. Dasabuvir and ritonavir were in the public domain, but the 3D regimen was under patent rights due to the associated drugs. Finally, simeprevir entered the public domain only when the regimen was no longer recommended.

## 7. Strategic public procurement of HCV products

As shown in Table 4, the MoH has negotiated much larger discounts since 2018, when it began using tenders instead of negotiating directly with manufacturers. The first tender (87/2018) was limited to sofosbuvir, with offers from Gilead and two distributors of Blanver’s generic: Hospinova and Maxima. Gilead opposed any competition, claiming that sofosbuvir was under patent exclusivity because the INPI had published an intention to grant the patent covering the intermediate, and several others were still pending. Gilead also claimed that the tender was unfulfillable and should be cancelled because the maximum price was impossibly low. Citing statements from the MoH and the INPI in a class action against the grant of the intermediate patent, Hospinova argued that Blanver used another intermediate and that pending applications cannot forbid sales, or else provisional rights would be the same as patent rights. In other words, it assumed the risk of selling the generic because it could circumvent any protection that sofosbuvir could obtain. Lastly, it accepted the maximum price as a one-off offer, given the urgency for HCV treatments. The auctioneer decided in favour of Hospinova, which was 63% cheaper than Gilead’s best offer.

**Table 4.** HCV centralised procurement history (2015–2024).

Product	Drugs	Lab	Type		2015	2016	2017	2018	2019	2020	2021	2022	2023	2024
Daklinza	daclatasvir	BMS	Originator	**$** **N**	12,143 21,834	10,057 30,317	9,183 10,460	9,047 14,760	- -	- -				

daclatasvir dihydrochloride	daclatasvir	Blanver	Generic	**$** **N**									-	-
													-	-
daclatasvir dihydrochloride	daclatasvir	Fiocruz	Generic	**$** **N**									4,398 7,738	-
														-
Zepatier	elbasvir/ grazoprevir	MSD	Originator	**$** **N**					- -	- -	- -			

Maviret	glecaprevir/ pibrentasvir	AbbVie	Originator	**$** **N**					6,048 25,118	- -	- -	- -	- -	-
														-
Harvoni	ledipasvir/ sofosbuvir	Gilead	Originator	**$** **N**					*5,940* *29,254*	*7,830* *10,336*	- -	- -		

Olysio	simeprevir	J&J	Originator	**$** **N**	12,455 8,772	10,013 5,076								

Sovaldi	sofosbuvir	Gilead	Originator	**$** **N**	32,738 31,956	20,637 35,056	18,508 12,715	- -	- -	- -	- -	- -	- -	-
														-
sofosbuvir	sofosbuvir	Blanver	Generic	**$** **N**				*3,616* *13,820*	- -	- -	- -	- -	- -	-
														-
sofosbuvir	sofosbuvir	Fiocruz	Generic	**$** **N**				-	- -	- -	- -	- -	2,359 7,738	-
								-						-
Sophir	sofosbuvir	Blanver	Branded generic	**$** **N**				- -	- -	- -	- -			

sofosbuvir	sofosbuvir	EMS	Generic	**$** **N**							- -	- -	- -	-
														-
sofosbuvir	sofosbuvir	Furp	Generic	**$** **N**							- -	- -	- -	2,329
														3,869
Epclusa	velpatasvir/ sofosbuvir	Gilead	Originator	**$** **N**					*6,605* *12,028*	*9,661* *7,763*	*8,941* *7,439*	*7,449* *9,333*	*7,100* *7,292*	*-*
														*-*
Viekira	3D	AbbVie	Originator	**$** **N**			8,429 25,096	- -						

Note: This table includes purchases until January 2024. For each purchase, I indicate the price and quantity of standard treatment courses. I updated prices to January 2024 using the Extended National Consumer Price Index, when the average exchange rate for the United States dollar was R$5.13 (BCB – Brazilian Central Bank, 2024; IBGE – Brazilian Institute of Geography & Statistics, 2023). Tenders are in italics; otherwise, direct negotiations. I report the year when purchase contracts were signed. Blank spaces indicate when products were not recommended for treatment or lacked marketing approval. Dashes indicate that products were not purchased in that year, despite being recommended and having marketing approval.

The second tender (105/2018) was the most competitive, including all regimens recommended at the time and attracting five manufacturers. Gilead won six groups, selling ledipasvir/sofosbuvir and velpatasvir/sofosbuvir. Instead of opposing Blanver’s participation, Gilead gave aggressive discounts on the combinations. While Blanver and BMS also gave significant discounts, Gilead’s offers were cheaper than sofosbuvir alone. AbbVie and MSD did not engage in this bidding war, placing offers at significantly higher prices. As a result, the MoH purchased treatments for six of the eight groups with a 52% discount on the maximum prices. No one won the other groups because all offers exceeded the maximum. Seven months later, the MoH negotiated a contract directly with AbbVie for glecaprevir/pibrentasvir, securing an 80% discount on its previous best offer, possibly influenced by the competition in the tender.

The third tender (56/2020) included all regimens except for sofosbuvir+daclatasvir. Only Gilead joined, placing offers for ledipasvir/sofosbuvir and velpatasvir/sofosbuvir. It claimed that the MoH used an incorrect exchange rate to estimate maximum prices and increased purchase volumes after publishing the call. Using the correct rate, Gilead’s offer was 2% cheaper in United States dollars than the previous tender, but 44% more expensive in Brazilian reais, and exceeded the maximum. The MoH raised the maximum to accommodate Gilead’s offer, given the need for treatments.

The fourth tender (161/2021) was designed for a small purchase of sofosbuvir alone. Despite this tender covering only 5% of the first tender’s purchase volume, the MoH set the same maximum price, to which Blanver had agreed only on a one-off basis. There was no purchase because Blanver submitted the only offer, and it exceeded the maximum.

The fifth tender (28/2022) focused on sofosbuvir+daclatasvir, velpatasvir/sofosbuvir and glecaprevir/pibrentasvir. Despite Blanver and Gilead placing offers, there was no effective competition. Blanver was disqualified when no offer was made for daclatasvir, since the drugs had to be bought together, and for listing prices per tablet, not treatment. Gilead offered velpatasvir/sofosbuvir at the same price as the third tender, which meant a real-term 7% discount in United States dollars. This was above the maximum price, which Gilead claimed had been estimated based on another regimen, but the MoH denied it. Gilead argued that Blanver should also be disqualified due to the grant of the intermediate patent. Again, the auctioneer rejected this appeal. Lastly, Gilead argued that the tender should not have included sofosbuvir+daclatasvir, as there was no version of daclatasvir on the market. The auctioneer replied that a generic daclatasvir was expected to be launched in time. As shown in Section 4, this happened in December 2022, after the tender. The tender ended without any purchase.

The sixth tender (78/2022) for velpatasvir/sofosbuvir and glecaprevir/pibrentasvir had only two sellers: Gilead and its distributor, Elfa. There was no effective competition since Elfa’s offer was twelve times higher than Gilead’s, which was 10% cheaper than its winning offer in the fourth tender, in United States dollars.

Since then, the MoH has negotiated three contracts with the public laboratories involved in PDPs, buying daclatasvir and sofosbuvir from Fiocruz in October 2023 and the same quantity of sofosbuvir for the same price from the Fundação para o Remédio Popular (Furp) in January 2024. These were great deals for sofosbuvir, 35% cheaper than the previous lowest price, negotiated in the first tender. However, the price of daclatasvir was 46% higher than BMS’s best offer in the second tender since Fiocruz’s product was imported (see Section 4). Thus, the price of sofosbuvir+daclatasvir was 14% higher than ledipasvir/sofosbuvir and 2% higher than velpatasvir/sofosbuvir in the second tender, which are still the two cheapest treatments the MoH has negotiated.

## 8. Discussion

Applying the matrix of four competition scenarios presented in Section 2, all direct purchases of HCV products in Brazil were cases of scenario A. The first tender was scenario B, involving the originator and distributors of the generic sofosbuvir. The second tender included all regimens, with multiple originators and one generic manufacturer, representing scenario D. All other tenders were instances of scenario A since the government effectively negotiated with only one seller.

The most significant discounts happened in the first and second tenders. The MoH has yet to negotiate prices lower than those in the second tender. One may see Gilead’s significant discounts in that tender as a short-term strategy to exclude competitors, especially Blanver, since subsequent sales were negotiated at higher prices. However, no subsequent tender had effective competition among originators and generic manufacturers, reducing the government’s bargaining power. In that sense, the longevity of this procurement strategy’s effects depends on the capacity to maintain higher levels of competition in tenders. Nonetheless, this may still be a useful policy tool, even if it yields only short-term savings. Comparing HCV drug prices across 65 countries between 2013 and 2019, Brazil had the second-largest price reduction as a percentage of gross domestic product (GDP) per capita, only behind France (Hudzik et al., 2023).

To discuss these results, lessons are drawn from the empirical literature. Generics tend to be considerably cheaper than originator products, with prices varying widely by country (Bonnifield et al., 2019; Wouters et al., 2019). In Brazil, most generics are at least 60% cheaper than originator products (Souza et al., 2023), and generic competition leads to a 28% reduction in minimum prices and an 8% reduction in originator products’ prices (Rocha, 2024). However, originator product prices may increase rather than decrease after generic entry if the originator’s market share is not challenged, in what is called the generics paradox (Regan, 2008). In addition, drug prices tend to be less affected by competition among patented drugs when substitutability is low or when few alternatives exist (Barrenho et al., 2023). Thus, scenario D should have the highest competition level, and scenario A the lowest. Future empirical studies with more data should investigate how scenarios B and C rank in the middle.

There is nothing new about using tenders to reduce drug prices via generic competition in off-patent tenders, as in scenario B (Maniadakis et al., 2018; Parmaksiz et al., 2022). Recently, some countries have begun exploring on-patent tenders with competition among originators of therapeutically comparable drugs, as in scenario C (Barrenho et al., 2023; Wouters et al., 2019). Even the industry has highlighted the benefits of these two types of tenders (Roediger et al., 2019). The novelty of the Brazilian strategy lies in having tenders with on- and off-patent competition simultaneously, as in scenario D.

Based on these findings, one may argue that the government can increase competition by using tenders under scenarios C and D. If a drug is under patent exclusivity, there is only one supplier, so it is reasonable to assume direct negotiation is the only procurement route. However, if drugs are highly comparable in effectiveness, the government differentiates them less and may simply buy the cheapest and maximise the purchase volume. Having at least one drug in the public domain, as in scenario D but not in C, increases the substitutability and strengthens this strategy by boosting the government’s bargaining power. Still, the government should never ignore differences in effectiveness to create the conditions for this strategy. Effectiveness should remain a technical assessment to ensure access to high-quality treatment.

Furthermore, this policy is influenced by the rigour of patent examination, the strength of opposition systems, and the approach to patent rights. It is not a coincidence that the most contentious drug, sofosbuvir, never obtained a compound patent in Brazil. Moreover, the Brazilian MoH, the INPI and the auctioneers never accepted Gilead’s claims that pending applications and the patent covering the chemical intermediate effectively blocked generic competition. The combination of these factors allowed the government to have tenders under scenarios B and D. In a country with a weak patent system, where most patents are granted without rigorous examination, purchases are restricted to scenarios A and C for much longer, involving only originators. However, that does not mean that patents should be intentionally rejected to allow generic competition. This case shows that rigorous examination leads to protection being granted to the specific elements that deserve it, which is essential to balancing public and private interests in the patent system (Andersen, 2004; Granstrand, 2009; Rockett, 2010).

Nevertheless, scholars and the industry have also pointed to the risk of tenders leading to market concentration (Barrenho et al., 2023; Maniadakis et al., 2018; Parmaksiz et al., 2022; Roediger et al., 2019; Wouters et al., 2019). Pharmaceutical tendering may render some market segments less attractive due to the significant discounts, especially under exclusivity. Indeed, AbbVie, BMS and MSD did not participate in any tender after the aggressive competition in the second. Three common recommendations are segmenting the tender to avoid a single-award system that could lead to complete failure, adopting a broader approach to the award criteria that combines price analysis with the overall economic cost of supply, and monitoring the supply to evaluate success beyond contract signing.

In addition, this study can only speak to the applicability of this strategy for drug tenders. While tenders are often used for other pharmaceutical products, such as vaccines (Parmaksiz et al., 2022), exclusivity often stems from factors other than patents, such as trade secrets and know-how, and the substitutability of biological products tends to be lower than that of therapeutics.

Many studies on HCV drug procurement in Brazil also stress the importance of PDPs in promoting local productive capabilities (Achcar & Fonseca, 2024; Fernandes et al., 2024). However, what led to generic registration only for daclatasvir and sofosbuvir was the combination regimen being recommended while the drugs lacked effective patent exclusivity (see Section 5). If these drugs had been under effective patent exclusivity, the MoH would have been limited to purchasing from the originators, despite having invested in the PDPs. The MoH even discussed establishing a PDP for sofosbuvir with Gilead, but some have suggested that Gilead was doing so to block the other PDPs in case no patent was granted, which it also did judicially (Achcar & Fonseca, 2024; Cassier & Correa, 2019). The considerable uncertainty about patent exclusivity led the MoH to suspend the sofosbuvir PDPs from 2018 to 2023, despite continuing to negotiate with Blanver. Still, Fonseca and Shadlen (2026, p. ii26) argue that ‘PDPs demonstrate how procurement can be used not only to meet health needs but also to foster industrial development, capacity, and greater equity.’ Therefore, promoting local productive capabilities can increase the likelihood of having tenders under scenarios B and D, but the PDPs have issues that should be addressed, especially regarding patent exclusivity.

Finally, some stress the importance of Anvisa’s price regulation (Salomão Filho & Ido, 2022). Notwithstanding their relevance for smaller purchases, price caps are too high to affect centralised procurement: the tenders’ maximum prices were, on average, 90% below Anvisa’s caps. The Administrative Council for Economic Defence (CADE) used a similar argument when refusing to proceed with a representation against Gilead for allegedly abusing its economic powers by raising the price of Sovaldi after the first sofosbuvir grant. CADE concluded that raising prices in small purchases while still below Anvisa’s caps is not anti-competitive, especially given the repeated discounts in centralised procurement (CADE – Administrative Council for Economic Defence, 2022). Some also argued that price caps are misaligned with market trends because they are not revised and are only adjusted to reflect inflation and industry-wide productivity changes (Souza et al., 2022).

## 9. Conclusion

This article investigated Brazil’s strategy of inducing competition in the centralised procurement of HCV drugs by having originators of alternative treatments compete among themselves and with generic manufacturers. Two main factors were identified as the drivers of this strategy’s success: (1) focusing treatment on regimens that were virtually equal in effectiveness led to a change in purchase rationale from cost-effectiveness to simply buying the cheapest product; and (2) the robust patent examination and opposition mechanisms and a nuanced approach to patent protection anticipated generic competition. As a result, originators and generic manufacturers of different drugs competed directly in the same tender, increasing the government’s bargaining power and reducing drug prices, which is essential to achieving the goal of eliminating HCV as a public health threat by 2030.

A matrix of four scenarios of patent exclusivity and therapeutic substitutability was developed to discuss this strategy. Because there were few purchases to analyse and they did not represent all scenarios, this article could not statistically determine the impact on the government’s bargaining power. Still, the results are in line with the literature on drug prices and competition in public procurement. The external validity of these findings was discussed in terms of the longevity of the gains in bargaining power and the types of pharmaceutical products being purchased. Furthermore, consideration was given to the fact that patent examination and the assessment of therapeutic substitutability should be determined technically and independently and should not be interfered with to produce the conditions for the type of tender here analysed. This is essential to promoting balanced patent stimuli and access to high-quality treatment.

This strategy was hindered by market concentration, whether due to fewer competitors or to their refusal to participate in tenders. Thus, the government should invest in developing local productive capabilities and making tenders more attractive for originators and generic manufacturers. These efforts should be helped by the introduction of two pangenotypic regimens: sofosbuvir/velpatasvir/voxilaprevir and sofosbuvir+ravidasvir. The former was registered by Gilead and incorporated into SUS, but has not yet been included in the guidelines or purchased (MoH – Ministry of Health, 2022b). The latter is an unpatented drug developed by the Drugs for Neglected Diseases Initiative (DNDi), which Pharco is transferring to Blanver and Fiocruz (DNDi – Drugs for Neglected Diseases Initiative, 2024; Douglass et al., 2018; Longínio dos Santos, 2023).

This article also discussed the influence of other public policies. While helpful, the PDPs were not the determinant of generic competition, and there are significant issues with the programme and its incentives. Notwithstanding the relevance of Anvisa’s price regulation, it did not affect centralised procurement because the caps were well above the negotiated prices. Future studies should analyse other diseases in which all competition scenarios are represented and investigate this strategy in contexts without local production policies or with smaller purchases.

As such, Brazil’s experience with centralised tenders for HCV drugs offers important lessons for increasing competition to expand access to high-quality treatments, based on the levels of therapeutic substitutability and patent exclusivity. Thus, governments should explore on- and off-patent tendering more frequently, invest in developing local productive capacities, make tenders more attractive, and review price regulation policies.

## Data Availability

The data that support the findings of this study are available from the corresponding author, EM, upon reasonable request.
